# Do Oral Pathogens Inhabit the Eye and Play a Role in Ocular Diseases?

**DOI:** 10.3390/jcm11102938

**Published:** 2022-05-23

**Authors:** Pachiappan Arjunan, Radhika Swaminathan

**Affiliations:** 1 Department of Periodontics, Dental College of Georgia, Augusta University, Augusta, GA 30912, USA; radhipachi2021@gmail.com; 2 James and Jean Culver Vision Discovery Institute, Augusta University, Augusta, GA 30912, USA

**Keywords:** ocular microbiome, oral microbiota, periodontal, inflammation, dysbiosis, homeostasis, age-related macular degeneration, glaucoma, diabetic retinopathy, uveitis

## Abstract

Fascinatingly, the immune-privileged healthy eye has a small unique population of microbiota. The human microbiome project led to continuing interest in the ocular microbiome. Typically, ocular microflorae are commensals of low diversity that colonize the external and internal sites of the eye, without instigating any disorders. Ocular commensals modulate immunity and optimally regulate host defense against pathogenic invasion, both on the ocular surface and neuroretina. Yet, any alteration in this symbiotic relationship culminates in the perturbation of ocular homeostasis and shifts the equilibrium toward local or systemic inflammation and, in turn, impaired visual function. A compositional variation in the ocular microbiota is associated with surface disorders such as keratitis, blepharitis, and conjunctivitis. Nevertheless, innovative studies now implicate non-ocular microbial dysbiosis in glaucoma, age-related macular degeneration (AMD), uveitis, and diabetic retinopathy. Accordingly, prompt identification of the extra-ocular etiology and a methodical understanding of the mechanisms of invasion and host-microbial interaction is of paramount importance for preventative and therapeutic interventions for vision-threatening conditions. This review article aims to explore the current literature evidence to better comprehend the role of oral pathogens in the etiopathogenesis of ocular diseases, specifically AMD.

## 1. Introduction

The Centers for Disease Control and Prevention reports that greater than 4.2 million Americans aged 40 years and above are either legally blind or have low visual acuity [1]. Despite the approved notion that the eye is an immune-privileged organ, and its microbiota is passive in the immune defense, there is a propagating interest in studying the potential interplay between the host and microbial ecosystem.

Microbiota collectively refers to all the microorganisms such as bacteria, virus, fungi, archaea, and protists that exists in a commensal, symbiotic, or pathogenic relationship with the host. Correspondingly, the microbiome refers to the genetic elements of the microbes living inside and on the skin, gut, and mucosal surfaces [2,3,4,5]. These microbes participate in several physiological processes ranging from digestion, synthesis of vitamins, regulation of inflammation and immune system, and more importantly, surface barrier protection against pathogenic invasion [6,7]. Notably, environmental factors, dietary habits, age, and genetic make-up strongly influence the microbiota, which, in turn, impacts health through direct or indirect strategies [8]. In normalcy, homeostasis is maintained through a cordial interaction between the host and the infinite number of resident microbes that populate the entire stretch of our body. Amassed scientific reports have emphasized the critical role of the microbiota and microbiome in human health and disease and have underscored that microbial dysbiosis contributes to the pathogenesis of lifestyle disorders, auto-immune, neuropsychiatric, and even neoplastic diseases [9,10]. Hence, the microbiota is regarded as the master key and clinical experts have embarked on the journey to explore and target the human microbiome to treat various eye diseases.

This review attempts to add more insights to our current understanding of the association between the pathogens of extra-ocular origin and eye diseases. Following a brief discussion of the ocular and oral microbiome, we have discussed the established and potential role of periodontal pathogens in the pathophysiology of eye diseases, particularly age-related macular degeneration (AMD).

## 2. Ocular Microbiome

The initial efforts at characterizing the core human microbiome disregarded the eye; however, there are now rigorous research efforts aimed at defining the ocular microbiome and emphasizing the concept of the “microbiota–gut–retina axis” [11,12,13]. The eyes are one type of organ that is constantly exposed to the environment and attracts diverse microorganisms. The tear film and mucin secretions protect the eye from foreign objects with their antimicrobial components, namely lysozyme, lactoferrin, and defensins, which prevent microbial colonization [14]. The conjunctiva and the cornea of the eye lodge the majority of the ocular microbiome. As evidenced by previous research efforts, relative to other microbiomes of the human hosts, which reside in the gut, mouth, nose, and skin, a healthy eye has a small unique, and expansive microbiome with low diversity [15,16,17]. Similar to other mucosal sites, the ocular microbiome plays a vital role in optimally regulating homeostasis and establishing host defense against pathogen invasion and its proliferation. The consortium of microbes inhabiting the eye includes bacteria, fungi, and viruses, while the bacterial groups are extensively focused. Among these, the fungi and viruses constitute less than 2% of the specimens [18,19]. Strikingly, tremendous research works and advancements in technologies (next-generation sequencing of 16S rDNA) have identified more than 500 bacterial genera from the conjunctival swabs [20]. The most abundantly cultivated bacterial organisms in a normal healthy eye are coagulase-negative staphylococci, *Acinetobacter*, *Methylobacterium*, *Propionibacterium*, *Pseudomonas*, *Streptophyta*, *Sphingomonas*, and *Corynebacterium* species [16,21].

Even though the eye has adopted a harmonious relationship with the commensals, a compositional variation in the ocular microbiota is associated with ocular surface disorders [22,23] and that of the non-ocular microbiome [24] in scleritis, glaucoma, diabetic retinopathy (DR) [25], uveitis [26,27,28,29], Retinitis Pigmentosa, Sjogren’s Syndrome, and AMD. A plethora of recent research has indicated the involvement of microbes of extra-ocular origin in the development and prognosis of ophthalmic pathologies (Table 1), which include bacteria, fungi, and viruses. It is noteworthy that more recent studies have identified the association of viral components in eye diseases such as uveitis in chronic infections of hepatitis B and hepatitis C viruses [30,31], cytomegalovirus in AMD [32,33], and glaucoma [34], and more interestingly, coronavirus (COVID-19) is also implicated in ocular diseases [35,36,37,38]. Several studies have determined that homeostasis of the ocular surface is directly or indirectly impacted by the complex intestinal microbiome [39]. In this regard, we intended to focus on the oral microbiome, especially the bacterial species that have been identified with a conspicuous role as a cofactor in the etiopathogenesis of a wide range of eye diseases.

## 3. Oral Microbiome

The neonatal human oral cavity, the first part of our digestive tract, is comparatively sterile and the acquisition of microbial species commences with the first feeding [45]. The oral cavity has the second-largest diverse microbial consortium following the gut and harbors over 700 species of bacteria [46]. The diverse microbial biofilms that live harmlessly regulating immune homeostasis are unique to different intraoral locations [47]. The periodontal tissue has the favorable architecture and environment for the microorganisms to be a successful inhabitant niche. Remarkably, the interaction between these microbial species within the oral community forms a crucial element in determining the pathogenesis of a multitude of oral (dental caries, periodontitis, endodontic infections, tonsillitis, and alveolar osteitis) and extra-oral diseases. These bacterial species produce opportunistic infections under altered settings, particularly poor oral hygiene, change in dietary habits, pH, host immune response, and genetic factors [48]. Periodontal disease (PD) is one such local disease that results from subgingival plaque accumulation and perturbation in the microbial community, which shifts the equilibrium from health to disease [49]. *Streptococcus gordonii* (*S. gordonii*) and *Streptococcus sanguinis* are the pioneer species critical in the initiation and progression of dental plaque formation by their ability to adhere to components of the salivary pellicle and other oral bacteria [50,51]. In the subgingival environment, *Porphyromonas gingivalis* (*Pg*), *Treponema denticola* (*T. denticola*), and *Tannerella forsythia* (*T. forsythia*) form a consortium as the prime “red complex” periodontopathogenic bacteria. *Aggregatibacter actinomycetemcomitans* (*A. actinomycetemcomitans*), *Fusobacterium nucleatum* (*F. nucleatum*), *Prevotella intermedia*, *Campylobacter rectus*, and *Eikenella corrodens* are other notable species involved in PD [52]. The red complex groups were most prevalent and strongly correlated with pocket depth and bleeding on probing, especially *F. nucleatum,* which was detected at high levels in all the samples [53].

In addition to the pathogenic effects in the oral cavity, the oral microbes often become certified disease-causing pathogens in immune-compromised subjects. The prototype periodontal pathogen *Pg* has been implicated in the pathogenesis of pancreatic cancers following *A. actinomycetemcomitans* [54]. A perturbed balance in the oral microbiome conglomerate culminates in a variety of chronic systemic disorders, including cardiovascular disease, stroke, preterm birth, diabetes, and pneumonia [55]. Oral dysbiosis especially through periodontal inflammation is linked to oral, esophageal, gastric, lung, pancreatic, prostate, and breast cancers [56]. During the 18th century, the “germ theory of disease” proposed by Louis Pasteur and Robert Koch and Miller’s “focal theory of infection” revolutionized the field of microbiology and listed a variety of diseases, namely septicemia, meningitis, pneumonia, tuberculosis, and syphilis, as having their origins from communication with the oral cavity. The inflammation of the tonsils, middle ear, lungs, meninges, and pericardium was also linked to oral infections [57]. In the late 1980s, epidemiological studies showed evidence linking oral dysbiosis [58] through periodontal diseases as a risk factor in a wide spectrum of systemic diseases. Offenbacher, in 1996, presented the concept of “periodontal medicine,” confirming this correlation in human and animal study models [59]. Sampaio-Maia et al. highlighted that the oral microbiome modifies the balance between health and disease, locally and systemically [60]. Moreover, the following studies hypothesized that the periodontal pathogens contribute to cardiovascular and respiratory disorders, diabetes mellitus, adverse pregnancy outcomes, neurodegenerative diseases, chronic kidney diseases, and osteoporosis, stretching the list to oral and extra-oral cancers. Therefore, activities of the oral microbiome may serve as potential biomarkers in the diagnosis of systemic diseases and in optimizing therapeutic targets.

Although the theory of oral foci of infection belongs to the prehistoric days, the link between periodontal disease and eye disorders has resurfaced lately. It is intriguing to note that the oral cavity function as the gateway for external microorganisms and those that inhabit the mouth and associated structures impact distant systems and organs such as the eye. As it is scientifically proven that many systemic diseases manifest in the oral cavity, it is also known that chronic inflammation originating from the oral cavity influence the pathogenesis of diseases at the systemic level [61]. Several latest studies have strongly suggested the existence of a gut-retinal axis, emphasizing the role of dysbiosis of gut microbiota in the inception and advancement of uveitis [62], AMD [63], and glaucoma [64]. Yet, as early as the 4th century, Hippocrates discussed a case of arthritis healing with the extraction of an infected tooth. In the following 90s, an outburst of research ensued, based on the association between periodontitis and systemic diseases [65,66]. It is noteworthy that the correlation between eye diseases and oral health is being reevaluated in late years through limited evidence. In this line, developing research intends to enlighten the involvement of oral microorganisms in the inception and prognosis of eye diseases, especially AMD and glaucoma. Chronic periodontal inflammation and the microbiome share various significant elements with many systemic conditions including pro-inflammatory mediators, bacterial metabolites, and genetic predisposition. Hence, a thorough analysis of the anomalous alterations in the mouth is vital for the meticulous identification and prevention of certain systemic diseases. The observation that the oral cavity is being recognized as the “diagnostic mirror” of the entire body has inspired the research arena to dissect the contribution of periodontal pathogens to the risk of ocular disorders [67]. Hence, through this review article, we have attempted to recapitulate the association and the potential role of periodontal pathogens in certain ophthalmic diseases.

## 4. Eye Diseases Associated with Oral Pathogens

Even before the advancement of sophisticated molecular biology techniques, eye diseases were regarded as manifestations of infections in the mouth. A few of the ancient studies interrelated the resolution of the given disease upon elimination of oral sepsis by extraction of the infected teeth [68]. In this context, bacteria and or their toxic metabolites and reflex nervous irritation were considered to be the crucial factors. In successive years, the concept of “oral foci of infection” lost its attention. Nonetheless, the latest studies successively acknowledged periodontitis as a risk factor in the pathogenesis of assorted inflammatory eye diseases including scleritis, iritis, glaucoma, diabetic retinopathy (DR), uveitis, retinitis pigmentosa (RP), and Sjogren syndrome (SS) (Table 2). In the recent period, AMD is being linked to oral health through hitherto undetermined mechanisms. We have attempted to discuss the most universally observed links between PD and eye disorders.

### 4.1. Scleritis

Scleritis is a vision-threatening chronic inflammation of the outermost coat of the eye caused by trauma, infection, or drug allergy [80]. Guncu et al. reported a case of periodontitis with symptomatic anterior diffuse scleritis [78] and noticed the resolution of scleritis following effective periodontal treatment. This report indicated that scleritis may be induced by increased systemic inflammation due to periodontal disease, and the resolution of scleritis was accomplished after the management of periodontitis, due to the reduction in the levels of inflammatory markers such as CRP and IL-6, as previously reported [81,82]. Scientific evidence shows that PD patients present with elevated inflammatory markers in their circulation, markedly cytokines IL-1 and TNF-alpha, PGE2, and hydrolytic enzymes [83,84]. It can be deduced that heightened systemic inflammation, rooting from the microbiota in the subgingival sites of the oral cavity, imposes a persistent bacterial challenge to the host periodontitis and plays a role in initiating scleritis.

### 4.2. Diabetic Retinopathy

**Figure 1:**
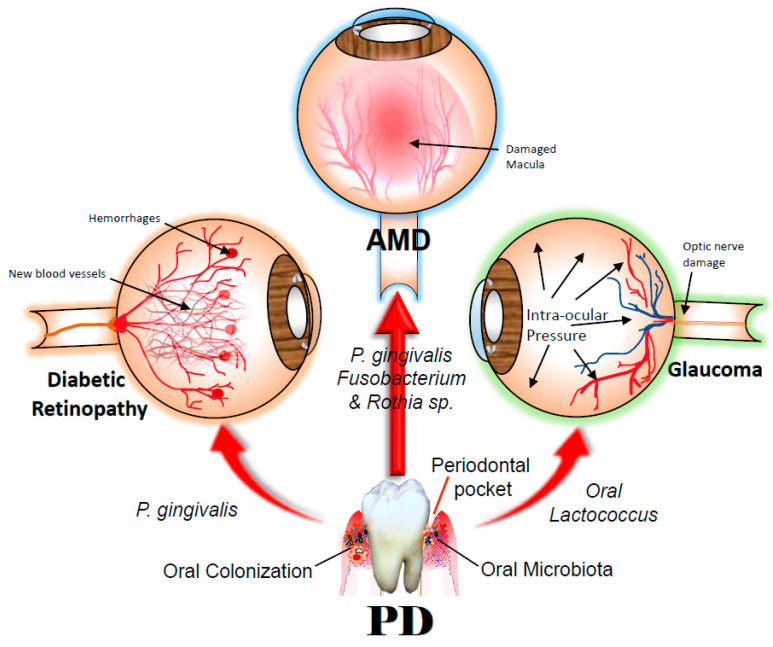
The link between periodontal pathogens and eye diseases. Growing scientific studies show a palpable association between oral pathogens (PD) and eye diseases (AMD, Glaucoma, and Diabetic Retinopathy). A mechanistic explanation for the association between chronic inflammaging diseases such as AMD and PD is an imperative need to derive preventive and therapeutic targets. Arjunan et al. in an in vitro study characterized *Porphyromonas gingivalis (Pg)* invasion in the human RPE cells and its prolonged survival by autophagy evasion within the RPE cells [70]. Another in vivo study employing a pioneering AMD + PD murine model demonstrated the role of periodontal infection in the augmentation of AMD phenotype [69]). *Fusobacterium* and *Rothia* species were identified as risk factors in the AMD by Pockpa et al., [72] and Rullo et al., [42], respectively. Chiu et al. demonstrated that *Pg* increases the risk for early diabetic retinopathy [98]), and oral *Lactococcus* was found to be markedly depleted in glaucoma patients, as shown by Yoon et al. [74]). AMD—Age-related Macular Degeneration; PD—Periodontal disease.

Diabetic retinopathy (DR) presents with severe damage to the microvasculature of the retina from complications of uncontrolled diabetes [43,85]. Investigations on the immune-modulatory responses to bacteria in diabetic subjects have been a subject of significant research interest for many years. In the same regard, studies have established that LPS acts as a strong stimulant for the release of cytokines, which are key inducers of insulin resistance [86]. The bacterial lipopolysaccharide (LPS) from *Escherichia coli* has been shown to exhibit major effects on insulin sensitivity [86,87]. Earlier, Bhat et al. showed that *Pg*-LPS has substantial implications on the development of pancreatic β-cell compensation and insulin resistance in prediabetics with PD [88]. Similarly, several studies have identified single-nucleotide polymorphisms (SNPs) within genes encoding for pro-inflammatory cytokines on periodontal health as important modifiers in PD and DR [89]. Other longitudinal studies have suggested elevated IL-6 and CRP levels associated with PD, as significant risk factors for insulin resistance and diabetes mellitus (DM) [90]. The phagocytic activities of neutrophils have been found to be compromised in diabetic patients, which leads to reduced bacterial killing and the augmented destruction of periodontal epithelial attachment [91]. In addition, the proinflammatory cytokines, lL-1β and TNF-α, produced in response to periodontal infection are responsible for insulin resistance and an imbalance in glycemic control in PD patients [91]. TNF-α and IL-1β levels were commonly elevated in the GCF of both PD and DM patients. The marked rise in IL-1β and TNF-α in response to bacteremia induces hyperlipidemia in addition to pancreatic β-cell destruction [92]. More importantly, in diabetic retinas, the levels of TNF-α were higher, which exacerbate the loss of pericytes and endothelial cells and plays a major role in the pathophysiology of DR [93]. Furthermore, periodontal therapy not only alleviated oral inflammation, but also reduced the systemic levels of IL6, TNF-α, and CRP [94,95]. In DM groups, scientists analyzed the gut bacterial composition and found a significant depletion of *Coriobacteriaceae, Veillonellaceae, Streptococcaceae*, and enrichment of *Burkholderiaceae* and *Burkholderiales* families [43,96]. Importantly, Chiu et al. hypothesized that exposure to *Pg* increases the risk for early DR [73,97] (Figure 1, Table 2). Cumulatively, per these confirmations, we can understand that periodontal pathogens have a bidirectional impact on diabetes and hence DR, the main complications of DM.

### 4.3. Glaucoma

Glaucoma, the second most common cause of blindness worldwide [99], is a neurodegenerative disease that affects the optic nerve, exclusively the retinal ganglion cells (RGCs) in the neural retina and their axons in the optic nerve. As a proven source of persistent chronic inflammation, the periodontal pathogens induce vascular alterations, resulting in activation of the local immune system within the retina and optic nerve head, allowing circulating immune or bacterial components to gain access to these sites. The immune mediators consecutively cause damage to the optic nerve cells by priming the local microglia after entering the retina and optic nerve site. Astafurov et al. in their pilot case-control study found that peripheral or extra-ocular bacterial activity could be potentially contributing to the pathogenesis of glaucoma [75,77] (Table 1). The oral bacterial load in patients with glaucoma was significantly higher than those without glaucoma, suggesting that glaucoma subjects are constantly exposed to higher levels of bacterial products (*p* < 0.017), which potentially exacerbate the severity and/or disease progression [74,75,76,77,99,100,101,102,103] (Figure 1). Moreover, low oral *Lactococcus* in the glaucoma population suggests that microbial dysbiosis could play an important role in glaucoma [74].

### 4.4. Sjogren’s Syndrome

In Sjogren’s Syndrome patients, diminished salivary gland secretion is the main trigger for increased bacterial dental plaque formation. Higher plaque values, sulcular bleeding, increased probing depths, attachment loss, and high periodontal indices were observed in oral examination among SS groups relative to healthy subjects. Similarly, compared to the control group, antibodies against *Streptococcus oralis* were significantly lower in SS patients. A higher antibody titer against *A. actinomycetemcomitans* and *Pg* was also revealed in these groups [104,105]. An investigation by Nayyar et al. in rats with chronic periodontitis revealed an increased expression of miRNA-155 in periodontal bacteria-infected gingiva and decreased expression of miRNA-155 in the submandibular salivary glands, along with identification of *Pg* and *T. denticola* [79]. This study indicates that primary periodontal infections can alter miRNA profiles in secondary sites such as the salivary glands and highlights the link between PD and SS. On the other hand, high levels of IL-17 in the plasma of patients with SS and elevated levels of IL-1β, IL-6, IL-23, and TGF-β in tissues affected by the disease determined the role of Th17 cells and IL-17 in the pathogenesis of this auto-immune disease [106]. Though the actual role of oral microbiota in the pathogenesis of SS is not thoroughly comprehensible, bacterial mimicry and metagenomic changes were identified to play a role in the onset of disease [44].

### 4.5. Age-Related Macular Degeneration

An association between oral health and susceptibility to ocular diseases such as AMD has obtained focus in recent years. AMD remains a prominent cause of irreversible central vision loss in aged populations of Western nations. AMD is a late-onset, asymptomatic, progressive eye disease affecting the macula and photoreceptor–retinal pigment epithelial complex crucial for normal vision. In the United States, approximately eleven million people are diagnosed with some form of AMD, and it is expected to double by 2050 [107,108,109]. The early-stage AMD affects >150 million and advanced AMD accounts for 10 million individuals [110]. Globally, the number of individuals affected by AMD was projected to reach two hundred million by the year 2020 and 300 million in 2040 [110,111,112]. Although age, genetic, environmental, metabolic, functional, and inflammation are documented primary etiologies, the pathophysiology of this complex asymptomatic disease is still unclear. With the unremitting progression of AMD, patients gradually lose their ability to perform daily routine activities, which strongly impacts the quality of life of affected ones. The current management strategies for this highly prevalent vision-threatening disease have many limitations without a permanent cure [110,113].

AMD presents in two main forms: (a) neovascular AMD (wet or exudative) and (b) geographic atrophy (dry or non-exudative AMD) [114]. Wet AMD causes central vision loss (central scotoma) as a result of the invasion of abnormal choroidal or retinal blood vessels and subsequent leakage of blood/fluid into the macula, which is the central area of the retina responsible for high-resolution color vision. The dry form, which affects 85% to 90% of AMD patients, is characterized by the gradual thinning and break-down of the macular tissue, resulting in irreversible destruction of the light-sensitive cells in the macula. In the asymptomatic preclinical phase of AMD, residues of undigested waste materials from the dysfunctional phagocytic cells accumulate in the space between the basement membrane (Bruch’s membrane) and the epithelial layer in the retina [115]. These deposits are referred to as “drusen,” the trademark of AMD pathology. These sediments are chemoattractant-rich and induce the degeneration of retinal pigmented epithelial (RPE) cells and subsequently the photoreceptor cells. A low-grade inflammatory response is incited by these deposits, leading to the recruitment of bone marrow-derived activated macrophages, overly critical in the induction of AMD pathology [116].

Owing to the multifactorial etiology of AMD, the pathophysiological mechanisms are not completely understood now. Nevertheless, an unlimited number of studies have emphasized the role of environmental, genetic, and nutritional factors (smoking, alcohol consumption, low dietary intake of antioxidants, omega fatty acids and carotenoids, high lipid levels), immune and vascular system interactions, autophagy dysfunction, and oxidative stress in the instigation and progression of AMD in vulnerable groups [117]. Epidemiological studies link a cholesterol-enriched diet and high plasma cholesterol levels with a high incidence of AMD [118]. While sharing the common hallmark features (drusen, β-amyloid peptide, oxidative stress, and apoptosis), AMD has lately been associated with Alzheimer’s disease [119]. Moreover, SNPs, mutations of mitochondrial DNA, and micro-RNAs have repercussions on the pathogenesis of AMD [120]. Noticeably, complement factor-H is categorized as a risk factor for AMD [121], where a shared gene locus advocates a probability that microbial interactions with the host complement trigger AMD via complement-mediated host cell damage [41]. Intriguingly, an alteration in the oral or nasal microbiome is identified to cause significant pathogenic effects. A case-control study analyzed the composition of nasal and oral microbiota in wet-AMD human patients and demonstrated local changes in the microbial composition and upregulation of pro-inflammatory pathways in distant sites such as those of the choroid-RPE complex [42]. Here, it is noteworthy that the impact of the extraocular microbiota on AMD pathogenesis is becoming registered these days (Table 1). In addition, through former studies, it is apparent that intestinal microbiota through their functional role in mucosal and systemic immunity contributes to the pathogenesis of AMD [62]. Zinkernagel et al. remarked that wet-AMD patients presented with gut microbiota that was enriched with *Anaerotruncus* spp., *Oscillibacter* spp., *Ruminococcus torques*, and *Eubacterium ventricose*, while the microbiota of healthy controls contained protective *Bacteroides eggerthii* [40,122]. Another study by Andriessen et al. showed that altered gut microbiota is related to the exacerbation of choroidal neovascularization (CNV) in predisposed obesity states and the development of neovascular lesions typical of AMD [123].

In the current era, the presence of aberrant chronic systemic inflammation in our body is recognized as a risk factor for degenerative diseases of the eye. Chronic oral inflammation caused by periodontal pathogens is being investigated for the potential risk and vulnerability in AMD patients with co-existing uncontrolled PD. Pockpa et al. emphasized that PD could be a potential risk factor for AMD and may play a role in the initial stages of AMD [72]. PD patients have presented with increased incidence for both nonexudative-type AMD (5.43 vs. 3.13 per 1000 person-years) and exudative-type AMD (0.52 vs. 0.28 per 1000 person-years) [124]. According to the latest case-control study, infections of specific combinations of periodontal microbiota, independent of PD, may be associated with AMD, and it may be concluded that oral microbiota has a critical role in eye health [98]. In a cross-sectional observational study, Shin et al. underscored the possibility of poor oral hygiene impacting the development of AMD in the Korean population. The middle-aged participants with severe PD were observed to be 1.61 times more likely to have AMD [125]. The National Health and Nutrition Examination Survey III in 2015 concluded that PD is independently associated with AMD in individuals aged 60 or younger. A total of 52.30% of individuals had PD with a prevalence rate of 11.45% for AMD [126]. Another study involving 56 AMD patients in the 45 to 90 age group indicated that the majority of the patients with lesions majorly involved the periodontium [127]. Karesvuo et al., in their population-based cross-sectional study, have presented the notion that alveolar bone loss could be independently associated with AMD specifically in the male population [128]. An increased intestinal permeability resulting from gut dysbiosis leads to chronic low-grade inflammation, characteristic of inflammaging, and elevated levels of IL-6, IL-1β, TNF-α, and VEGF-A, leading to the exacerbation of pathological angiogenesis [123]. Moreover, studies have reported elevated plasma levels of CRP, an acute-phase reactant, and an active regulator of the innate immune system as high-risk factors associated with advanced AMD [129,130]. Here, excessive levels of CRP are implicated in a variety of systemic diseases and are strongly associated with PD. Cumulative literature evidence pronounces the physiological link between periodontal pathogens and AMD, with limited mechanistic vindication for this novel link.

In this scenario, Arjunan et al. first reported the plausible mechanisms underlying the association between chronic inflammation as in PD and AMD [131] (Table 2). As a firsthand study, it demonstrated the role of intracellular periodontal bacterium, *Pg* in the AMD pathophysiology [131]. *P. gingivalis* is a renowned keystone species that impact the host directly by tissue damage and indirectly by hijacking the immune system to enhance their survival and multiplication [132]. Lately, this team indicated that invasion of RPE cells by *Pg* and its mutant strains upregulated AMD-related genes involved in angiogenesis, immunosuppression, and complement activation [70,133] (Figure 1). This specific analysis underscored the high invasive potential of *Pg*, which is enabled through autophagy dysfunction, which, in turn, attributes to the link between oral dysbiosis and ocular diseases [70]. Preceding studies have reported significant evidence correlating the levels of local and systemic biomarkers to the pathogenesis of both PD and AMD. This points to the notion that a dynamic interaction of mixed-species biofilm in the dental plaque and host innate immune system elicits inflammatory responses in extra-oral sites. A wide spectrum of investigations has identified altered immune pathways and genes of the innate immune system in the pathogenesis of AMD [134,135]. The degenerating RPE cells in the retina and choroidal tissue exacerbate chronic inflammation, along with age-related immune changes contributing to the existing destructive process [136]. Hence, it is advocated that a chronic low-grade oral inflammation sustained by dysbiosis of the oral cavity and a leaky attachment apparatus exclusively by the periodontal pathogens is attributed to the development of AMD.

### 4.6. Hypothesis Supporting the Association of Periodontal Pathogens and AMD

Through these years, extensive in vitro and in vivo evidence has revealed the atypical role of immunity in the development of AMD, attributed to the recruitment of immune cells, especially macrophages, inflammatory molecules, complement activation, or triggering the microglial cells. Owing to the former interpretations, three plausible mechanisms are conceivable by which periodontal infection potentially affects ectopic organs such as eyes: (a) transmigration of periodontopathogens; (b) microbial toxins; and (c) oral-hematogenous spread of pro-inflammatory mediators and antibodies [137].

(i) With the progression of PD, the healthy epithelium of the richly vascularized periodontal framework ulcerates as a result of proteolysis and exposes the underlying connective tissues and blood capillaries to the dental biofilm comprising multiple bacterial species [138]. Regular day-to-day procedures such as eating, brushing, and dental procedures cause the introduction of oral microbes into the bloodstream and lymphatics [139]. This condition referred to as bacteremia is polymicrobial, specifically with high numbers of anaerobic Gram-negative bacilli [140]. Under a healthy status, host defenses eliminate those microorganisms disseminated into the systemic circulation, counteracting the transient bacteremia. However, under compromised circumstances, the microorganisms disseminate to extraoral sites where they adhere to and invade wide-ranging tissues [141,142]. DNAs of the red-complex group, *A. actinomycetemcomitans* and *C. rectus*, have been recovered from atherosclerotic plaques and aneurysmal thrombus. *Pg*, the primary colonizer of the epithelium, has been detected in the postmortem brain tissue of AD patients [143]. *F. nucleatum* found ectopically in the gut is associated with human colorectal cancers, liver abscesses [144], appendicitis [145], mastoiditis, tonsillitis, and maxillary sinusitis [146]. Both *F. nucleatum* that belongs to the orange complex and *Pg* are linked to various types of Oro-digestive cancers owing to their tissue invasion capacities. Moreover, the composition and shifts in the nasal and oral microbiota were illustrated in wet AMD cases compared to controls without retinal diseases. The members of the *Actinobacteria* phyla, *Rothia* genus, *Propionibacteriaceae* family, and the *Corynebacteriaceae* genus are notable species retrieved from the oral cavity of AMD human subjects [42]. A multitude of reports has shown that periodontal pathogens evade the host defense by employing their seasoned strategy, which is host cell invasion. These species often use host proteins and enzymes to access and invade the cell. Distinctly, the adhesion and proliferation of periodontal bacteria is never confined to the oral cavity but extends to distant organs. The leakage and dissemination of LPS endotoxins released by the Gram-negative *Pg* is an important determinant of focal infections and extra-oral complications (atherosclerosis, myocardial infarction) [147]. The LPS-containing microvesicles function as “micro bullets” and fortify the invasive ability of *Pg,* thereby amplifying the destruction of periodontal tissues [148]. Following the diffusion of cytolytic enzymes and LPS into the bloodstream, TNF-α, IL-1β, prostaglandin E2 (PGE2), and interferon (IFN)-γ are released into the circulation, causing a systemic inflammatory burden [149]. Thus, translocation of these highly invasive microbes ensues in the development of protracted systemic inflammation and adverse host inflammatory responses.

(ii) Hypo or hyper-responsiveness of the immune system results in persistent damage to the periodontal tissue. A delicate balance between the microbial trigger and host immune response is significant and proportional to the severity and progression of PD. Chronic infection of the periodontium and a perpetual upregulation of pro-inflammatory mediators contributes to a systemic sequel with adverse effects. *Pg* is equipped with a unique virulency competently to destruct the host tissues and modify the immune system to support their survival and multiplication [132]. Markedly, these species camouflage from the host immune surveillance and survive for an extended period within the invaded tissues. Following dissemination and bacteremia, oral bacteria or their soluble products react with the circulating antibodies and form macromolecular complexes. As per the “Toxic-infective theory” of Rosenow [150,151], these immunocomplexes contribute to acute and chronic inflammatory responses at distant body sites [136] such as the synovial joint, choroid of the eye, kidney, and skin. This observation has been reported in ocular inflammatory diseases such as Behcet’s syndrome, uveitis, chronic urticaria, and Crohn’s disease [152]. As noted by a wide range of investigations, periodontium acts as the reservoir of many proinflammatory cytokines, especially TNF-α, IL-1β, IFN-γ, and PGE2, which are implicated in an array of systemic disorders [142]. Intriguingly, a growing body of studies underscored the manifestation of pro-inflammatory factors and low-grade inflammation in the maturing retina correlating with AMD pathophysiology. A series of scientific evidence has established that the chronic aberrant inflammatory response aids the progression of AMD into advanced stages, culminating in the irreversible decline of visual function. Indisputably, low-grade chronic inflammation provoked by oral pathogens has a significant impact on vulnerable distant body sites such as the eyes.

### 4.7. Our Viewpoints on the “Oro-Optic Network”

A congregation of basic and clinical studies has expounded the biological association of chronic periodontal inflammation with an array of systemic diseases; however, mechanistic clarifications for the linkage between periodontal pathogens and ocular diseases are due for analysis. While referring to the scientific proposition that periodontitis incites aberrant inflammation, which radiates into the systemic health of vulnerable patients, we approach the potential link in the “Oro-optic network” in terms of anatomical proximity and ectopic immunological responses.

### 4.8. Proximity of Anatomical Structures

Many decades ago, animal research considered unnoticed dental ailments contributing directly or indirectly to vision-threatening eye disease. Ocular manifestations of oral diseases were appreciated in dogs and cats where the anatomical proximity between posterior maxillary (upper back) teeth and the orbit was correlated [153], although no direct human cases were presented with this scenario. The human eye is shown to maintain an immune-privileged state [154,155] such as the equivalent brain. Nonetheless, despite the notion that the entry of immune cells into the eye is nonexistent [156], recent studies have shown that certain immune cells are recruited to the eye following retinal injury through infections or inflammation, as noticed in several models [157]. As illustrated before, the anatomic accessibility of oral microflora to the bloodstream via the ulcerated periodontal epithelium facilitates bacteremia and the systemic spread of bacterial components, their metabolic wastes, and immune macro-complexes. In this context, the “danger triangle of the face” (small imaginary triangle extending from the corners of the mouth to the nasal bridge, direction, and pattern of blood supply to the nose, venous drainage from the facial veins, and pterygoid plexus) obligates a remark as significant anatomical landmarks. It is because an infection traveling from this specific area, specifically the upper jaw, eyes, and nasal region, spread into the cavernous sinus, resulting in a fatal condition called cavernous sinus thrombosis. We hypothesize that this anatomical juxtaposition may be related to the metastatic dissemination of tissue-invasive periodontal pathogens, especially *Pg* and/or their bi-products, to the orbital structures through blood or lymphatics, where they feasibly provoke aberrant host immune responses.

### 4.9. Immunological Perspective

The end-product of crosstalk between the host and microbial community results in local and systemic complications in the PD environment. Scientific underpinnings are signifying that the systemic inflammatory burden prompted by the periodontopathogen infection occurs enigmatically either through the subversion of host defenses and/or augmentation of inflammatory responses in the body. Innate immunity plays an elemental role in chronic inflammation, and periodontal pathogens trigger innate immunity through the activation of TLRs that result in the production of pro-inflammatory cytokines and recruitment of macrophages, granulocytes, and dendritic cells along with lymphocytes into the inflammatory zone. *Pg* has evolved unique mechanisms to circumvent the host immune response by employing strategies to survive, sustain, and persist within the oral tissues, particularly the antigen-presenting dendritic cells (DCs), for a prolonged period [70,158]. Studies have discovered *Pg* surviving actively within the macrophages [159]. The major and minor fimbriae of *Pg* are the key elements in the disruption of immune homeostasis as the fimbrial adhesins of *Pg* facilitate biofilm formation, invasion, and dissemination through blood DCs. Numerous scientific findings have highlighted that the internalization of *Pg* produces a “privileged niche” under a dormant state while insulating them from host immune surveillance. It should be appreciated that periodontal bacteria internalized in monocytes, macrophages, or the DCs at the diseased site operate a “Trojan horse” modus, to disseminate to other tissues [160]. It is already established that *Pg* efficiently subverts normal DC function and transmutes it to a highly migratory immunosuppressive phenotype [160]. This aids in the metastatic spread from the oral sites to a remote site as observed in atherosclerosis. Several animal experiments have demonstrated that *Pg* is well-formed to colonize distant organs such as a coronary artery, placenta, liver, pancreas, and, as lately found, invading brain tissues. Undeniably, this potent microbiome contained within the blood-DCs of patients with PD has profound effects on systemic health [161].

### 4.10. How about the Retina?

In light of these novel discoveries, our focus is being directed to the healthy retina, in which the RPE cells play a key role in immune responses against overt inflammation with the help of TLRs. The initial recruitment of monocyte-derived macrophages ensuing immune activation is required to process the byproducts from the photoreceptors and RPE. Under normal healthy settings, RPE offers immune suppression through tight junction-mediated barrier integrity and anti-inflammatory cytokines, while the retinal microglia provide additional immune surveillance by clearing the cell debris. The microglia are the resident inflammatory cells of the retina as with those tissue macrophages and microglia in the brain. To purge the byproducts from visual activities and to maintain normal vision, the subretinal physiological migration of microglia is obligatory, while its impairment instigates the death of photoreceptor cells and exacerbates retinal degeneration [114]. The disparity in the process of elimination of damaged tissue filtrate leads to the accumulation of drusen. Under healthy status, a balanced supply of oxygen and metabolic substrates, as well as an intact BRB, are key requirements for the maintenance of retinal structure and function [162]. Notably, RPE is a common site for inflammatory assault, which leads to the breakdown of the barrier functions and choroidal neovascularization (CNV). Functionally, the blood–retinal barrier (BRB) preserves the physiological environment of the neural retina and limits inflammatory responses, being dependent on the integrity of the RPE [163]. Significantly, RPE is the main target for many neurodegenerative diseases such as AMD [164]. In wet AMD, the breakdown of the BRB allows circulating immune cells to access a highly immunogenic environment, resulting in macrophage recruitment. Subsequently, these macrophages initiate a neovascular response and produce an abnormal, leaky vascular network, causing fluid leakage followed by the development of fibrosis. The dry form presents with areas of degenerated RPE and photoreceptors with the release of toxic inflammatory mediators and cytokines. The role of macrophages has been described in CNV in terms of polarization concerning pro-inflammatory (M1 macrophages) and pro-angiogenic (M2 macrophages) responses [114]. As per Wagley et al.’s hypothesis, the pathophysiological pathway of the pathogen-associated molecular pattern (PAMP) recognition and the subsequent triggering of the immune response and tissue destruction via molecular mimicry is considerable in this infection-driven inflammatory model for the association of PD with AMD [126].

Other studies have established *Pg* access into immature DCs in situ in diseased human gingiva [165]. It is identified that these infected DCs, exclusively through the DC-SIGN (DC-specific intercellular adhesion molecule-3 grabbing nonintegrin receptor) sustain an immature state and remain highly resistant to apoptosis. Typically, matured DCs engage in the expansion and differentiation of T-cells that regulate or suppress other immune T-cells, whereas the immature DCs that reside in the mucosa are destined to harness different allergens and antigens, which, in turn, stimulates their maturation. In human gingiva, the maturation of DCs results in downregulated antigen-capture machinery while upregulating costimulatory molecules, cytokines (interleukin-1β, IL-6) antigen-presenting molecules (MHC I and II), and adhesion molecules (ICAM-1, VLA4), which are necessary to prime the naive T-cells in lymphoid organs [165,166].

Arjunan et al. elucidated that the dysbiotic periodontal pathogen *Pg* in high concentrations efficiently invades RPE cells, replicates, and sustains within them [70]. Based on recent data, one can hypothesize that invasion and autophagy evasion by this keystone species could be contemplated as one of the contributing elements in the pathogenesis of retinal degenerative diseases such as AMD. In relevance to this perception, the lysosome/vacuolar escape or survival mechanism of *Pg* strains might occur through the deactivation of autophagy-signaling molecules in RPE cells. The host–pathogen interaction and the strategies by which RPE cells respond to the monomicrobial or polymicrobial biofilm and its pathological impact in vitro and in vivo has remained elusive. Consecutively, the same team has demonstrated a role for periodontal pathogens in the augmentation of the AMD phenotype in vivo by employing a groundbreaking AMD + PD murine model [69]. This report identified particularly the role of the keystone periodontopathogen *Pg* in the progression of neovasculogenesis in a laser-induced choroidal-neovascularization (Li-CNV) mouse retina. Multiple inflammatory drusen-like lesions, reduced retinal thickness, and increased vascular leakage were the significant findings reported in the AMD + PD mice retinae. They have also identified augmented expression of oxidative stress, angiogenesis, and pro-inflammatory mediators, whereas antioxidants and anti-inflammatory genes were notably declined. Further, interestingly, the key finding of the study documented is the expression of *Pg* and its fimbrial 16s-rRNA gene in the AMD + PD mice retinae [69,71]. Given this setting, the authors postulate that upon stimulation by the dysbiotic oral pathogens or through systemic diffusion of their metabolic by-products, the inflammatory immune cells may function as potential carriers of the oral pathogens and disseminate them to distant body sites including the immune-privileged sites such as the eye. However, upcoming investigations in human subjects will resolve the puzzle in verifying the causal role of *Pg* and other oral pathogens in the invasion of RPE cells and their interaction with the intraocular immune system in patients with AMD and PD.

## 5. Summary and Future Directions

A logical exploration of the mechanisms involved in host–bacteria interaction is mandatory to understand the pathophysiology of infectious, inflammatory, autoimmune, and neoplastic ocular diseases. The previous and latest research efforts have clearly distinguished the association between oral microbiota and systemic health; nonetheless, there are a few imperative concerns that remain unaddressed in the context of eye diseases. Specifically, in AMD, researchers have only partial clarifications from murine models for how the oral commensal or periodontal pathogens enter the intraocular space and immune-privileged sites of the retina; secondly, the molecular mechanisms of the cell–cell or cell–receptor interaction and host responses to direct and indirect pathogenic stimulation; and thirdly, whether PD is a biomarker for the susceptibility of AMD in humans. Our perspective based on the accrued literature evidence is that the diverse microbiota dispersed throughout the body may be highly influential, through direct or indirect strategies, on both native and ectopic organs and contributes to a wide array of diseases. Hence, the prime objective now is to ascertain the palpable link between the pathogenesis of ocular diseases and periodontal microbiota and the effects of its resolution in preventing or remedying eye diseases such as AMD in human subjects. Through additional evidence-based studies, elucidation of the specific mechanisms involved in the transmigration of oral bacteria to extraoral sites such as the eye, and characterization of the regulatory mechanisms of the oral and ocular microbiological ecosystem will open new prospects in the field of infection-driven inflammatory eye diseases and add valuable insights to preventive and therapeutic applications.

## Figures and Tables

**Table 1:** Extra and Intra Ocular Pathogens and Diseases.

Ocular Disease	Extra-Intra Ocular Pathogens	Microbial Levels in Various Eye Diseases/Disorders *	Reference
AMD Neovascular AMD	*Prevotella Ruminococcaceae, Rikenellaceae* (intestinal microbiome)	High Low	Lin et al., *Curr. Opin. Ophthalmol.*, 2018 [24].
	*Anaerotruncus, Oscillibacter, Ruminococcus torques, Eubacterium ventriosum, Negativicutes (Firmicutes species)*	High	Zinkernagel et al., *Sci. Rep.*, 2017 [40].
	*Gemella, Streptococcus* (pharyngeal microbiome)	High	Ho et al., *PLoS ONE*, 2018 [41].
	*Streptococcus, Burkholderiales*	High	Ho et al., *PLoS ONE*, 2018 [41].
	*Actinomycetaceae, Gemella, Proteobacteria, Actinomyces, Veillonella* (nasal microbiome)	High	Rullo et al., *Sci. Rep.*, 2020 [42].
	*Cytomegalovirus* (CMV)	Ocular human CMV latency could be a significant risk factor for the development of AMD	Xu et al., *J. Pathol.*, 2020 [32]. Research article
Diabetic Retinopathy	*Bifidobacterium, Lactobacillus*	High	Huang et al., *Front. Cell. Infect. Microbiol*., 2021 [43].
	*Escherichia-Shigella, Faecalibacterium*, *Eubacterium, Clostridium* genera	Low	
Glaucoma	*Bacteroides, Prevotella*	High	Baim et al., *Exp. Biol. Med*., 2019 [25].
	*Cytomegalovirus*	Larger cup-to-disc ratio, more severe corneal endothelial cell loss, and greater iris depigmentation in CMV-positive patients	Fan et.al., *BMC Ophthalmol*., 2022 [34].
	*Varicella zoster virus* (VZV)	VZV-AUSG (anterior uveitis secondary glaucoma) presented with a higher IOP and worse visual acuity.	
Keratitis (Bacterial and Fungal)	*Bacteroides fragilis, Dorea, Shigella, Treponema*	High (Fecal samples)	Jayasudha et al., *PLoS ONE*, 2018 [27].
Uveitis	*Prevotella copri*	High	Kalyana Chakravarthy et al., *Indian J. Microbiol*., 2018 [28].
	*Bacteroides species*	Low	
	*Hepatitis B (HBV) and hepatitis C virus (HCV).* (Cohort study)	Uveitis is mostly associated with HBV.	Kridin et al., *Eye*, 2022 [30]. Research article
	*HBV and HCV.*	Patients with HBV and HCV coinfection had the highest risk of uveitis.	Tien et al., *Retina*, 2016 [31]. Research article
Sjogren Syndrome	*Pseudobutyrivibrio, Escherichia, Shigella, Blautia, Streptococcus parabacteroides, Fecalibacterium, Prevotella, Bacteroides*	High	Trujillo-Vargas, C. M., et al., *Ocul.* *Surf.*, 2020 [44].
		Low	
	*Coronavirus* (SARS-CoV-2)	SARS-CoV-2 infection shares symptomatology and morphological landmarks with Dry Eye Disease and diabetic neuropathy.	Barros et.al., *Ocul Sur*., 2022 [38]. Research article

* Increased (high) or decreased (low) levels of bacteria/virus identified in different disease specimens obtained from saliva, tongue, mucosa, and feces.

**Table 2:** Oral pathogens in ocular diseases.

S.no	Eye Disease	Oral Pathogens	Major Findings	References
1.	AMD	*Porphyromonas* *gingivalis (Pg)*	First in vivo study examining the role of periodontal infection in augmentation of AMD phenotype, with the aid of a pioneering AMD + PD murine model.	Arjunan et al., *Antioxidants*, 2021 [69] (New). Research article (in vivo)
		*P. gingivalis*	Invasion of human ARPE cells by *Pg* and its prolonged survival by autophagy evasion within these cells, significant (*p* < 0.01) downregulation of autophagosome complex-related genes.	Arjunan et al., *Sci. Rep.*, 2020 [70]. Research article (in vitro)
			Oral dysbiosis plays a role in the induction and/or progression of inflammatory eye diseases.	Arjunan et al., *Int. Rev. Immunol*., 2021 [71]. Review article
		*Fusobacterium species* *Propionibacteriales*, *Rothia*, *Staphylococcus*, *Cornyebacteriaceae*	Periodontitis may be a plausible risk factor for AMD.	Pockpa et al., *Perm. J*., 2019 [72], Review article
			Shifts in communities of oral and nasal bacteria. Oral microbes identified: *Burkholderiales* (7.41 log2 fold change, *p* = 3.29 × 10^−5^), *Actinomyceataceae* (6.22 log2 fold change, *p* = 3.73 × 10^−6^), and *Gemella* (5.28 log2 fold change, *p* = 0.0002). Fusobacterium (*p* = 1.00 × 10^−10^).	Rullo et al., *Sci. Rep*., 2020 [42], Research article (Clinical study)
2.	Diabetic Retinopathy	-	Prevalence of retinopathy was much higher in diabetic subjects with periodontal disease than in those without it (15.1% vs. 7.8%, *p* < 0.001).	Horikawa et al., *Exp. Clin. Endocrinol. Diabetes*, 2019 [73]. Questionnaire Survey
3.	Glaucoma	-	low oral *Lactococcus* in the glaucoma population suggests that microbial dysbiosis could play an important role in glaucoma.	Yoon et al., *JOM*, 2021 [74], Research article
			Patients with periodontitis exhibited a higher risk of primary open-angle glaucoma (POAG).	Sun et al., *Sci. Rep*., 2020 [75]. Research article
			Number of teeth and alterations in the volume of oral bacteria are associated with glaucoma pathology.	Polla et al., *J. Glaucoma*., 2017 [76]. Case-control study
			Chronic subclinical peripheral inflammation and activation of microglia in the retina and optic nerve, mediated through TLR4 signaling and complement upregulation contributes to glaucomatous pathology.	Astafurov et al., *PLoS ONE*, 2014 [77], Research article
4.	Scleritis	-	Resolution of scleritis after periodontal treatment.	Guncu et al., *Eur. J. Dent*., 2011 [78]. Case report.
5.	Uveitis—Behcet’s Disease	*Streptococcus sanguinis (Ss)*	Differences in salivary or gut microbiome composition can trigger innate-derived inflammation.	Leccese et al., *Front Immunol*., 2019 [29]. Research article
6.	Sjogren Syndrome	*P. gingivalis,* *Treponema denticola (Td)*	Relative expression of miRNA-155 was increased in periodontal bacteria-infected rat gingiva. Primary periodontal infections can alter miRNA profiles in secondary sites such as the salivary gland and pancreas.	Nayar. G et al., *Anaerobe*, 2016 [79]. Research article

## Data Availability

Not applicable.
